# Urinary Extracellular Vesicles for Non-Invasive Quantification of Principal Cell Damage in Kidney Transplant Recipients

**DOI:** 10.3390/biom14091124

**Published:** 2024-09-05

**Authors:** Per Svenningsen, Rima Maslauskiene, Yaseelan Palarasah, Inga A. Bumblyte, Martin Tepel

**Affiliations:** 1 Department of Molecular Medicine, University of Southern Denmark, 5230 Odense, Denmark; psvenningsen@health.sdu.dk (P.S.);; 2 Department of Nephrology, Lithuanian University of Health Sciences, 44307 Kaunas, Lithuania; rima.maslauskiene@lsmu.lt (R.M.); ingaarune.bumblyte@lsmu.lt (I.A.B.); 3 Department of Nephrology, Odense University Hospital, 5000 Odense, Denmark

**Keywords:** exosomes, extracellular vesicles, damage of principal cells, urinary extracellular vesicle, aquaporin-2 abundance, allograft injury, kidney transplant

## Abstract

The objective of the present study was to compare principal cell-specific aquaporin-2 (AQP2) abundances in urinary extracellular vesicles (uEVs) on the first postoperative day in deceased-donor kidney transplant recipients without and with acute kidney injury. We measured uEV markers (CD9 and CD63) and the abundances of proximal tubular sodium-glucose transporter 2, distal tubular sodium/chloride cotransporter, and principal cell-specific aquaporin-2 using Western blotting of urine. uEV-AQP2 levels were normalized to living donor controls. The validation cohort consisted of 82 deceased-donor kidney transplant recipients who had a median age of 50 years (IQR 43 to 57 years). A total of 32% of recipients had acute kidney injury. The median uEV-AQP2 was significantly higher in recipients with acute kidney injury compared to immediate allograft function (2.05; IQR 0.87 to 2.83; vs. 0.81; IQR 0.44 to 1.78; *p* < 0.01). The Youden index indicated a uEV-AQP2 threshold of 2.00. Stratifying uEV-AQP2 into quartiles showed that recipients with higher uEV-AQP2 levels had higher rates of acute kidney injury (Cochran–Armitage, *p* = 0.001). The discovery cohort showed elevated CD9, CD63, and uEV-AQP2 levels in urine from recipients with acute kidney injury compared to immediate allograft function. We were able to quantify the damage of principal cells after kidney transplant to predict acute kidney injury using uEV-AQP2.

## 1. Introduction

Acute kidney injury is a severe complication after kidney transplantation. Acute kidney injury is observed in up to 40 percent of deceased-donor renal transplant recipients [1,2]. Acute kidney injury prolongs patient hospitalization and increases morbidity and health care costs [1,2]. The post-transplant development of acute kidney injury is multi-factorial, involving characteristics from kidney donors and recipients [3,4,5,6,7,8]. Acute kidney injury after deceased-donor kidney transplantation has been attributed to ischemia–reperfusion injury, and experimental models indicate that ischemia–reperfusion injury is associated with an increased release of urinary extracellular vesicles [9,10].

Urinary extracellular vesicles (uEVs) are membrane-bound organelles secreted from epithelial cells lining the urogenital system and contain molecular cargo, e.g., proteins, metabolites, and nucleic acids from their parental cells [11,12]. Epithelial-expressed proteins from all major tubular segments have been detected in uEVs, providing an opportunity for non-invasive access to intra-renal injury. Both in vitro and experimental studies suggest a correlation between cellular and EV levels of proteins [13]. Hypoxia is a potent stimulus for EV secretion and may result from high reactive oxygen species production during transplantation [14,15]. Hence, there is a high biological plausibility that determining segment cell-specific uEV abundances quantifies acute damage after kidney transplant. The objective of the present study was to compare principal cell-specific aquaporin-2 (AQP2) abundances in uEVs on the first postoperative day in deceased-donor kidney transplant recipients without and with acute kidney injury.

## 2. Materials and Methods

### 2.1. Study Design, Discovery and Validation Cohorts, and Ethical Statements

The discovery cohort samples were prospectively collected from the ongoing Molecular Monitoring after kidney transplantation (MoMoTx) study at Odense University Hospital, Denmark. Details from the “MoMoTx” study were published previously [6,7]. The study protocol was in accordance with the ethical standards of the Declarations of Helsinki and Istanbul, and its registration identifier at ClinicalTrials.gov is NCT01515605. The local ethics committee approved the study (Den Videnskabsetiske Komite for Region Syddanmark, Projekt-ID: 20100098). Written informed consent was obtained from all patients before entry into the study. Exclusion criteria were age below 18 years or missing consent. The discovery cohort comprised 16 deceased-donor kidney transplant recipients (median age 48 years, IQR 43 to 63 years). Eleven recipients (69%) were male. Seven recipients (44%) had acute kidney injury, whereas nine showed immediate allograft function. The validation cohort comprised kidney transplant recipients from deceased donors after brain death transplanted at the Lithuanian University of Health Sciences. Kidney transplant recipients from ABO-blood-type-compatible living donor transplants also transplanted at the Lithuanian University of Health Sciences served as controls. The study protocol was in accordance with the ethical standards of the Declarations of Helsinki and Istanbul. The local ethics committee approved the study (Kaunas Regional Biomedical Research Ethics Committee, Approval BE 2-9, 1 April 2014). Details from the cohort were published previously [16]. 

Baseline characteristics of donors and recipients and information on organ procurement were prospectively obtained from medical records. Induction therapy, immunosuppressive therapy, and concomitant medications were all made by the clinicians at the institution according to the local protocol. The standard immunosuppressive regime consisted of basiliximab or thymoglobulin, tacrolimus, mycophenolate mofetil, and methylprednisolone.

### 2.2. Isolation of Urinary Extracellular Vesicles

We isolated uEVs from samples from the discovery and the validation cohorts following the recommendations of the Urine Task Force of the International Society for Extracellular Vesicles [17]. Urine samples from the cohorts were collected from 6 h to 24 h post-transplant and stored at −80 °C until processed. Urine samples were thawed on ice in the presence of a protease inhibitor (https://www.sigmaaldrich.com/DK/en/product/sigma/p8340, accessed on 23 August 2024), vortexed, and centrifuged at 5000× *g* for 10 min at 4 °C. The supernatants were collected, and 16% polyethyleneglycol (PEG)-6000 and 1 M NaCl were added as previously described [18]. The solution was mixed briefly and incubated at 4 °C. Following overnight incubation, the uEVs were pelleted by centrifugation at 5000× *g* for 10 min at 4 °C and resuspended in Radioimmunoprecipitation assay buffer (RIPA buffer). The loading amount of uEVs was normalized to urinary creatinine as previously described [19].

### 2.3. Urinary Creatinine

Urinary creatinine concentration was determined with Creatinine 120 CP (ABX Pentra, Horiba ABX) on micro-lab 300 (Vital Scientific, Horiba ABX, Montpellier, France).

### 2.4. Western Blotting

Samples were mixed with 4× Sample Buffer and 10× Reducing Agent (Thermo Fischer Scientific, Lillerød, Denmark) and incubated for 5 min at 95 °C. Samples for Western blots of CD63 were not reduced. Samples were subjected to SDS-PAGE on self-cast Tris-HCl polyacrylamide gels (8–12% (Bio-Rad, Herlev, Denmark)) together with a size marker (Precision Plus Protein Dual Color (Bio-Rad, Herlev, Denmark)) and 1× Tris/Glycine/SDS Running buffer (ThermoFisher Scientific, Lillerød, Denmark). Proteins were blotted to a polyvinylidene difluoride (PVDF) membrane (Immobilon transfer membrane, Millipore, Denmark). The membrane was blocked for one hour in 5% dry skim milk in TBST (20 mM Tris-HCL, 137 mM). After additional washes with Tris-buffered saline with 0.1% Tween 20 detergent (TBST), it was probed with primary antibodies and detected with horseradish peroxidase (HRP)-conjugated secondary antibodies (DAKO, Santa Clara, CA 9505, USA) diluted 1:2000 in 3% milk-TBST. Protein bands were detected with enhanced chemical luminescence (Merck Søborg, Denmark) and visualized using the ChemiDoc XRS+ system (Bio–Rad Laboratories, Copenhagen, Denmark). AQP2, CD63, sodium-glucose transporter 2 (SGLT2), and sodium/chloride cotransporter (NCC) were detected using anti-AQP2 (sc-9882, Santa Cruz Biotechnology, Santa Cruz, CA, USA), anti-CD63 monoclonal antibody (cat. no. M1544, Sanquin, Amsterdam, The Netherlands), rabbit anti-SGLT2 (cat. no. 14210, Cell Signaling, Dambers, MA, USA), and rabbit anti-NCC (HPA028748, Sigma-Aldrich, Waltham, MA, USA) antibodies, respectively. Densitometry of the Western blots was performed using Fiji as described [20].

### 2.5. Production and Cloning of Monoclonal Anti-Aqp2 Antibodies

Two peptides corresponding to human AQP2 aa 33–40 [NWPQALPS] and aa 108–122 [TPADIRGLAVNALS] were synthesized (Genescript). Peptide coupled to diphtheria toxin and mixed with Freunds’ incomplete adjuvant was injected into BALB/c mice subcutaneously three times at a 14-day interval. Three days before the fusion, the mice received an intravenous injection with 30 μg antigen administered with adrenalin. The spleen was removed, and the splenocytes were fused with the SP2/0-AG14 myeloma cell line with polyethyleneglycol (PEG). Clones producing cell-conditioned medium reacting with the immunogenic peptides were selected, and cloning was performed by limited dilution. Single clones were grown in culture flasks in RPMI + 10% FCS. The mAbs were purified from culture supernatant by protein A affinity chromatography using the Äkta FPLC system according to the manufacturer’s instructions (Amersham Pharmacia, Uppsala, Sweden).

**Figure 1:**
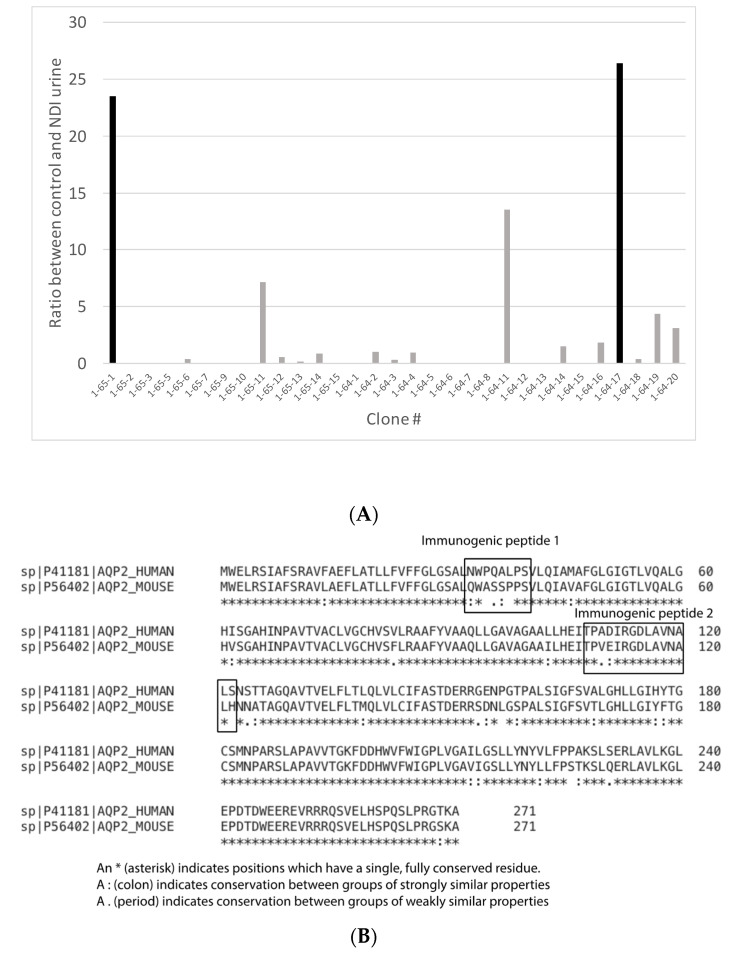

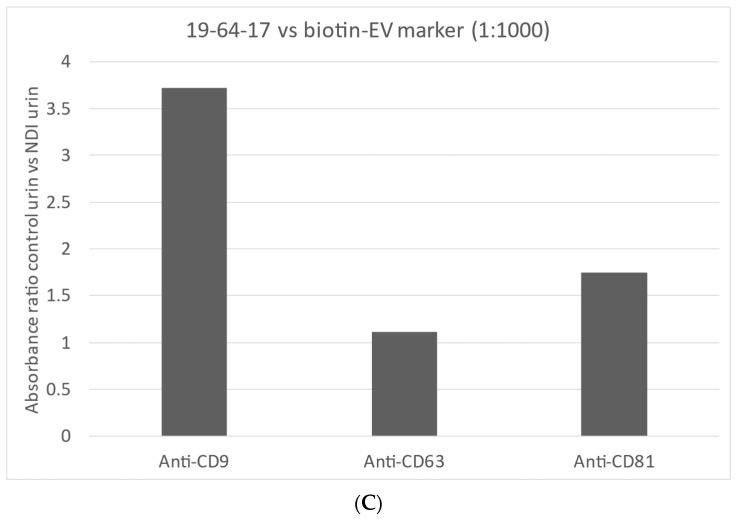
Development of monoclonal antibodies against human aquaporin-2 (AQP2). (**A**). The monoclonal antibodies were raised against the external epitopes of human AQP2, which are indicated with a rectangle. (**B)**. Screening of individual clones for their signal ratio in control and NDI uEVs. The clones showing the highest signal (black bars) were selected, i.e., clones 19-64-17 and 19-65-1. (**C**). Signal determination with different monoclonal capture antibodies. Anti-CD9 capture antibodies produced the highest signal compared to anti-CD63 and anti-CD81.

AQP2 remains in its native orientation in the EV membrane, i.e., the cytosolic side is inside the EV; therefore, we developed monoclonal antibodies against extracellular AQP2 epitopes (Figure 1A). We obtained 35 clones producing peptide-specific antibodies that were screened for their ability to capture uEVs (Figure 1B). The captured uEVs were detected with an anti-CD63 monoclonal antibody. In urine samples from healthy persons, the two antibodies 19-64-17 and 19-65-1 produced high absorbance values. The tetraspanins CD9, CD63, and CD81 are present in EVs, albeit in different abundances, dependent on the parental cell type [21]. Using specific monoclonal antibodies against the EV markers, we found that anti-CD9 produced the highest signal compared to anti-CD63 and anti-CD81 when used as detection antibodies (Figure 1C). Thus, antibodies 19-64-17 and 19-65-1 directed against two different extracellular epitopes in the human AQP2 protein, when combined with an anti-CD9 antibody, enable the analysis of uEV-AQP2 abundances.

### 2.6. Enzyme-Linked Immunosorbent Assay (ELISA)

Maxisorp plates (Nunc) were coated with 100 µL purified monoclonal anti-AQP2 antibodies (19-64-17 or 19-65-1) in phosphate-buffered saline (PBS) overnight at 4 °C and blocked with 0.5% bovine serum albumin (Sigma Aldrich, Darmstadt, Germany) in PBST. After washing in PBS, 200 µL urine was added and incubated for 1 h at room temperature. The plate was washed in PBS, and 100 µL anti-CD9-biotin (156-030, Ancell, Bayport, MN, USA) diluted 1:1000 in PBS was added and incubated for 1 h at room temperature. After washing in PBS, 100 µL streptavidin-HRP (Dako, Glostrup, Denmark) diluted 1:1000 in PBS was added and incubated for 1 h at room temperature. The plate was washed twice in PBST, and TMB (Sigma Aldrich) was added. After incubation for 15 min at room temperature, stop solution was added, and absorbance was measured.

### 2.7. Outcome Variables

The primary outcome variable was acute kidney injury after a kidney transplant. According to United Network for Organ Sharing, acute kidney injury was defined as the need for dialysis within the first week after transplantation [22,23]. Need for dialysis was considered by the treating physicians according to local guidelines and the best medical care after transplantation. Treating physicians were unaware of the levels of uEVs. The need for dialysis within the first week after transplantation was confirmed with a chart review. Immediate allograft function was defined as a routine outcome after transplantation without needing a dialysis session within the first week post-transplant.

### 2.8. Statistical Analyses

Continuous data are presented as the median and interquartile range (IQR). Frequency counts were calculated for categorical data. For continuous variables, a non-parametric Mann–Whitney test was performed. We performed receiver operating characteristic (ROC) curve analysis to detect the accuracy of principal cell-specific AQP2 abundance in uEVs to predict acute kidney injury after transplant. Positive and negative predictive values were calculated. Data were analyzed using GraphPad Prism software (version 6.0, GraphPad Software, La Jolla, CA, USA). All statistical tests were two-sided. Two-sided *p*-values less than 0.05 were considered to indicate statistical significance.

## 3. Results

### 3.1. Identification of uEVs in Post-Transplant Urine in the Discovery Cohort Using Western Blotting

**Figure 2:**
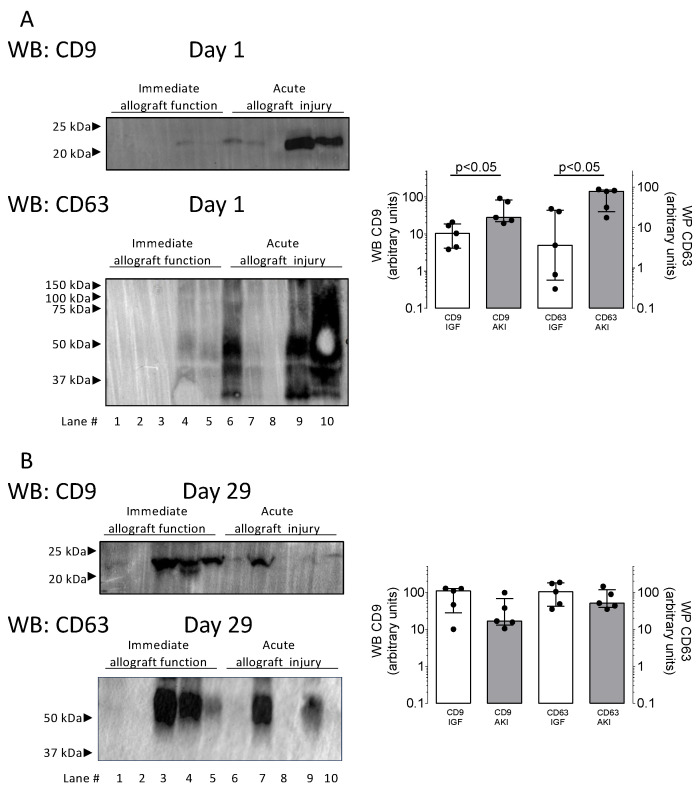
Representative Western blots of the expression of specific markers of extracellular vesicles, tetraspanin CD9 (molecular weight 24 kD), and tetraspanin CD63 (molecular weight 53 kD). Representative Western blots from 10 kidney transplant recipients with immediate allograft function (IGF, 5 samples) and acute kidney injury (5 samples) are shown, where postoperative urine had been obtained on day 1 (upper panels (**A**)) and day 29 post-transplant (lower panels (**B**)). Molecular weight markers are indicated. For quantification, densitometry of the Western blots was performed using Fiji 2. Groups were compared using the non-parametric Mann–Whitney test. (Original Western Blot Images see Supplementary Materials).

The clinical and biochemical characteristics of the discovery cohort are shown in Table 1. A Western blot analysis of uEVs showed increased abundances of uEV-specific tetraspanin CD9 and tetraspanin CD63 in patients with acute kidney injury on the first postoperative day (Figure 2A; *p* < 0.05 by Mann–Whitney test). On day 29 post-transplant, the CD9 and CD63 abundances were not different between patients with acute kidney injury and immediate allograft function (Figure 2B).

### 3.2. Comparison of uEV Abundance from Several Epithelial Sources in the Allograft

**Figure 3:**
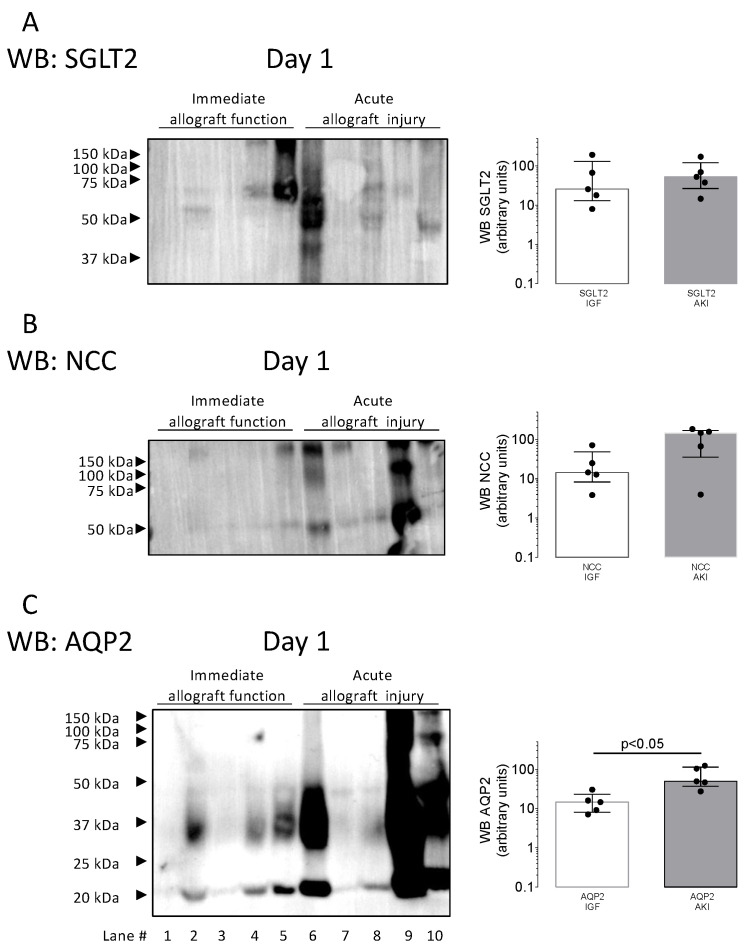
Representative Western blots of the expression of proximal tubular expressed sodium-glucose transporter 2 (SGLT2, upper panel), distal tubular expressed sodium/chloride cotransporter (NCC, middle panel), and principal cell-specific aquaporin-2 (AQP2, lower panel) in urinary extracellular vesicles (uEVs) obtained in post-transplant urine from kidney transplant recipients. Representative Western blots from 10 kidney transplant recipients with immediate allograft function (IGF) and acute kidney injury are shown, where urine was obtained on the first postoperative day. Molecular weight markers are indicated. For quantification, densitometry of the Western blots was performed using Fiji 2. Groups were compared using the non-parametric Mann–Whitney test. (Original Western Blot Images see Supplementary Materials).

To determine the cellular source of the increased uEV abundance in the patients with acute kidney injury, we analyzed the uEV abundance of proximal tubular expressed sodium-glucose transporter 2 (SGLT2), distal tubular expressed sodium/chloride cotransporter (NCC), and principal cell-specific AQP2. uEV-SGLT2 abundances (Figure 3A) and uEV-NCC abundances (Figure 3B) were not different between patients with acute kidney injury and immediate allograft function. However, uEV-AQP2 (Figure 3C) abundances were significantly higher in patients with acute kidney injury (*p* < 0.05 by Mann–Whitney test). The high uEV-AQP2 abundance prompted us to develop a high-throughput ELISA assay to detect uEV levels of AQP2 in the validation cohort.

### 3.3. Characteristics of Kidney Transplant Recipients in the Validation Cohort

In the validation cohort, we screened 108 recipients with incident deceased-donor kidney transplantation. To ensure detection of cellular damage in the validation cohort as early as possible, 82 urine samples that had been collected during the period from 6 h to 24 h post-transplant were available. Forty-three transplant recipients were male (52%), and the median age of the recipients was 50 years (IQR 43 to 57 years). The clinical and biochemical characteristics of the included deceased-donor kidney transplant recipients in the validation cohort are given in Table 2. All deceased-donor kidney transplant recipients obtained maintenance therapy with calcineurin inhibitors, mycophenolate mofetil, and methylprednisolone.

### 3.4. Acute Kidney Injury after Transplantation

According to the United Network for Organ Sharing, acute kidney injury was defined as dialysis within the first week after transplantation [22,23]. Acute kidney injury was observed in 29 of the 82 kidney transplant recipients (32%). Compared to the recipients with immediate graft function, the recipients with acute kidney injury had a higher prevalence of cardiovascular disease (34% vs. 15%; *p* = 0.05 by Fisher’s exact test), higher plasma creatinine before transplant (884 µmol/L; IQR 729 to 1053 µmol/L vs. 730 µmol/L; IQR 614 to 885 µmol/L; *p* = 0.01), and a significantly longer median cold ischemic time (960 min; IQR 780 to 1140 min; vs. 806 min; IQR 720 to 960 min; *p* = 0.02 by Mann–Whitney test). As indicated in Table 2, other characteristics, including the age of the recipient and dialysis vintage, were not different between the groups.

### 3.5. AQP2 Abundance in uEVs Determines Acute Kidney Injury Post-Transplant

**Figure 4:**
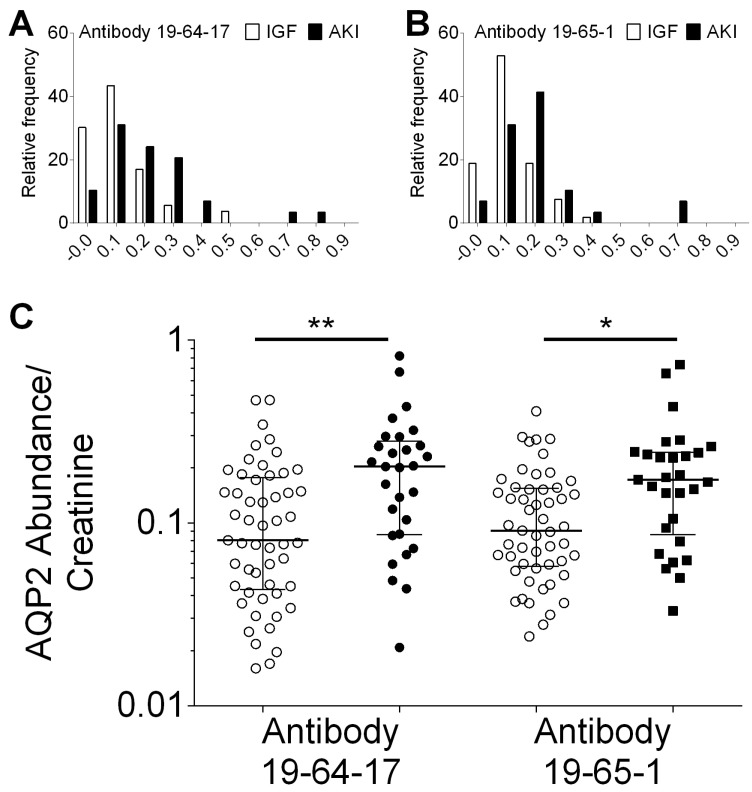
Abundance of principal cell-specific aquaporin-2 (AQP2) in urinary extracellular vesicles (uEVs). Graph showing the distribution of AQP2 abundance in uEVs from kidney transplant recipients with immediate allograft function (open bars) and acute kidney injury (AKI, filled bars). (**A**). AQP2 antibody 19-64-17. (**B**). AQP2 antibody 97-65-1. (**C**). Principal cell-specific AQP2 abundance in uEVs from kidney transplant recipients with immediate allograft function (open circles) and acute kidney injury (filled circles). ** *p* < 0.01; * *p* < 0.05 by Mann–Whitney test.

We tested all urine samples with antibody 19-64-17 and antibody 19-65-1 in independent assays. The median principal cell-specific AQP2 abundance in uEVs from kidney transplant recipients was 0.12 (IQR 0.06 to 0.20) using antibody 19-64-17 and 0.13 (IQR 0.06 to 0.18) using antibody 19-65-1. Next, we compared the abundance of principal cell-specific AQP2 in uEVs from kidney transplant recipients without and with acute kidney injury. The distributions of principal cell-specific AQP2 abundance in uEVs in these groups using specific antibody 19-64-17 and antibody 19-65-1 are depicted in Figure 4A. Compared to immediate allograft function, the abundance of AQP2 was shifted to higher levels in kidney transplant recipients with acute kidney injury. As shown in Figure 4B, AQP2 abundance using specific AQP2 antibody 19-64-17 was significantly higher in kidney transplant recipients with acute kidney injury (median 0.20; IQR 0.09 to 0.28) compared to immediate allograft function (median 0.08; IQR 0.04 to 0.18; *p* < 0.01 by Mann–Whitney test). AQP2 abundance using specific AQP2 antibody 19-65-1 was significantly higher in kidney transplant recipients with acute kidney injury (median 0.17; IQR 0.09 to 0.24) compared to immediate allograft function (median 0.09; IQR 0.06 to 0.15; *p* < 0.05 by Mann–Whitney test).

### 3.6. AQP2 Abundance in uEVs Indicates Damage of Principal Cell Post-Transplant

**Figure 5:**
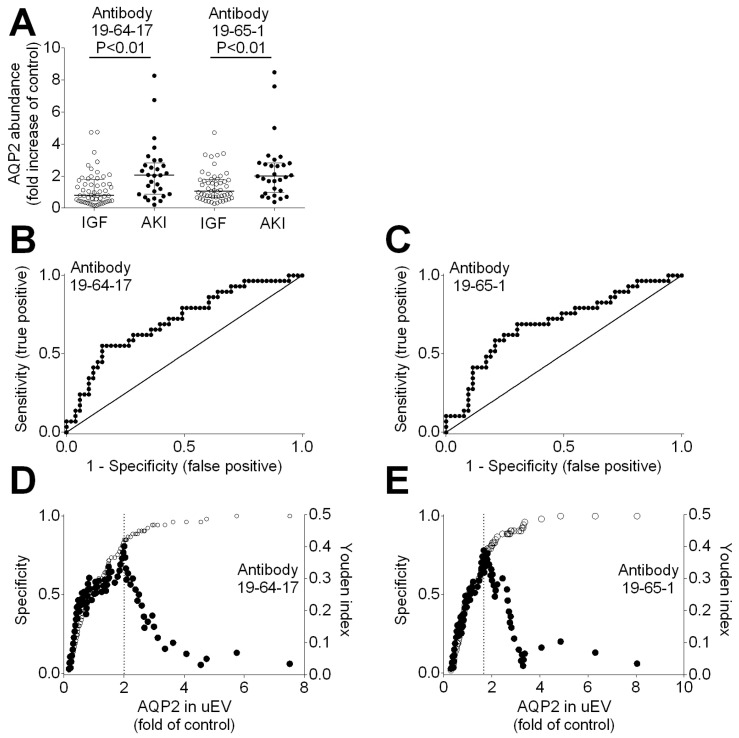
Principal cell-specific AQP2 abundance in urinary extracellular vesicles (uEVs) from deceased donors normalized to living donors with immediate allograft function as controls. (**A**). AQP2 abundance in uEVs from kidney transplant recipients with immediate allograft function (open circles) and acute kidney injury (AKI, filled circles). Left panel, specific AQP2 antibody 19-64-17; right panel, specific AQP2 antibody 97-65-1. Receiver operating characteristic (ROC) curve for prediction of acute kidney injury from AQP2 abundance in uEVs using antibody 19-64-17 (**B**) and antibody 97-65-1 (**C**). Graph showing specificity (open circles) and Youden index (filled circles) of AQP2 abundance in uEVs (fold increase of control) to predict acute kidney injury using antibody 19-64-17 (**D**) and antibody 97-65-1 (**E**).

We reasoned that principal cell-specific AQP2 abundance in uEVs obtained from three incident kidney transplants from living donors with immediate allograft function might serve as controls. Hence, to quantify damaged principal cells in kidney transplants from deceased donors, we calculated the ratio of principal cell-specific AQP2 abundance in uEVs from deceased donors and living donors with immediate allograft function. As shown in Figure 5A, using antibody 19-64-17, the median ratio of principal cell-specific AQP2 abundance in urinary extracellular vesicles was significantly higher in deceased-donor recipients with acute kidney injury compared to immediate allograft function (median 2.05; IQR 0.87 to 2.83; vs. median 0.81; IQR 0.44 to 1.78; *p* < 0.01 by Mann–Whitney test). When using antibody 19-65-1, we observed similar results: the median ratio of principal cell-specific AQP2 abundance in urinary extracellular vesicles was significantly higher in deceased-donor recipients with acute kidney injury compared to immediate allograft function (median 1.99; IQR 1.00 to 2.82; vs. median 1.05; IQR 0.67 to 1.79; *p* < 0.01 by Mann–Whitney test). Receiver operating characteristic (ROC) curves showed that principal cell-specific AQP2 abundance in uEVs predicted acute kidney injury (antibody 19-64-17; area under curve 0.71; 95% confidence interval 0.60 to 0.83; *p* = 0.001; Figure 5B; and antibody 19-65-1; area under curve 0.70; 95% confidence interval 0.58 to 0.82; *p* = 0.003; Figure 5C). The specificity and Youden index of principal cell-specific AQP2 abundance to predict acute kidney injury using AQP2 antibody 19-64-17 are shown in Figure 5D, and for AQP2 antibody 19-65-1 in Figure 5E, respectively.

From the Youden index, we determined a threshold value of 2.00. A total of 16 out of 82 recipients (20%) with an AQP2 abundance in uEVs greater than 2.00 compared to the controls showed acute kidney injury. In contrast, only 8 out of 82 recipients (10%) with an AQP2 abundance in uEVs of less than 2.00 compared to the controls showed acute kidney injury (*p* = 0.0003 by Fisher’s exact test). The sensitivity was 0.67 (95% CI 0.45 to 0.84), the specificity was 0.78 (95% CI 0.65 to 0.87), the positive predictive value was 0.55 (95% CI 0.36 to 0.74), and the negative predictive value was 0.85 (95% CI 0.72 to 0.93). The high negative predictive value indicates that if the abundance of principal cell-specific AQP2 is lower than two-fold the abundance in living donors, one can expect immediate allograft function.

**Figure 6:**
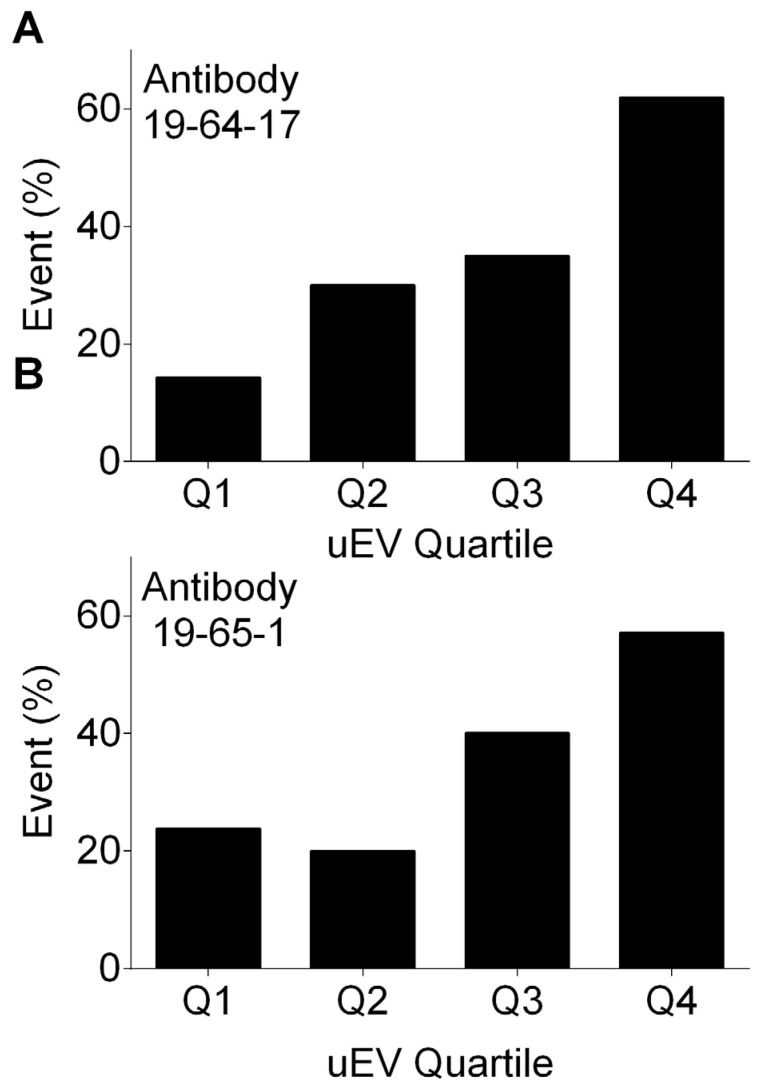
Cochran–Armitage test of trend between quartiles of abundance of aquaporin-2 in urinary extracellular vesicles (uEV-AQP2) and percentage of transplant recipients who experienced acute kidney injury in the validation cohort. The Cochran–Armitage test showed *p* = 0.002 and *p* = 0.01 when using 19-64-17 antibodies (**A**) and 19-65-1 antibodies (**B**), respectively.

When stratifying uEV-AQP2 into quartiles, we observed a statistically significant trend, where recipients with higher uEV-AQP2 levels showed higher rates of acute kidney injury. As indicated in Figure 6, the Cochran–Armitage test showed *p* = 0.002 and *p* = 0.01 when using 19-64-17 antibodies and 19-65-1 antibodies, respectively. Sensitivity analyses indicated that higher uEV-AQP2 levels showed higher rates of acute kidney injury in 29 recipients who were anuric prior to transplant (*p* = 0.018) as well as in 53 recipients with diuresis prior to transplant (*p* = 0.049). The effect was more pronounced in 34 female kidney donors (*p* = 0.006) than in 48 male kidney donors (*p* = 0.061).

## 4. Discussion

The present study quantified the damage of principal cells in kidney allografts early on the first postoperative day by determination of the abundance of principal cell-specific aquaprorin-2 (AQP2) in urinary extracellular vesicles (uEVs). We observed that if the principal cell-specific AQP2 abundance was lower than two-fold the abundance in living donors, one could expect an immediate allograft function. Principal cells are very important in the pathogenesis of acute kidney injury because they express high levels of aquaporins, which are important to reabsorb water.

There are several genuine advantages of investigating principal cell-specific AQP2 in uEVs after kidney transplant to quantify the damage of principal cells. First, uEVs resemble the molecular content of the parent cells from which they have been released [24]. Second, uEVs carry cell-specific markers from the segment of the nephron where they originated [25]. Third, uEV contents are protected against exogenous proteinases and RNases by their surrounding plasma membrane during urine passage [26,27]. Forth, uEVs can be obtained non-invasively, preventing the risks of invasive allograft biopsies. Fifth, experimental studies in cultured cells showed a direct correlation between cellular protein abundance and uEVs [9]. Last, the amount of uEVs has been attributed to ischemic reperfusion injury [12,28].

The recent literature indicates that the abundance of cell-specific proteins in uEVs is associated with cellular damage. Compared to allografts from living donors, acute kidney injury is more frequently observed in allografts from deceased donors. Therefore, the median principal cell-specific AQP2 abundance in uEVs in recipients with living donor transplantation with immediate allograft function was set to 1.00. Our results showed that the median ratio of principal cell-specific AQP2 abundance in uEVs was significantly higher in deceased-donor recipients with acute kidney injury than in immediate allograft function. That result aligns with the notion that increased damage of principal cells contributes to clinically relevant complications after transplant. We observed a high negative predictive value indicating that if principal cell-specific AQP2 abundance is lower than two-fold the abundance in living donors, one can expect an immediate graft function. Notably, recipients with acute kidney injury had a significantly longer cold ischemic time than recipients with immediate allograft function. Therefore, these findings may indicate that principal cell-specific AQP2 abundance in uEVs represents the extent of allograft damage leading to a clinically significant complication.

Our present study is in line with previous findings using uEVs in animal models: Koh et al. reported that the release of uEVs increases under hypoxic conditions [29]. Studies in rats showed that changes in uEVs’ transcript and protein abundance are quantitatively representative of the changes in the renal cortex after toxic damage [27]. They also showed that a two-fold increase in transcript expression in the renal cortex was paralleled by a two-fold increase in uEVs after puromycin administration [27]. Changes in EV-AQP2 levels were described in animal models of renal ischemia–reperfusion injury [6]. Protein markers of uEVs (for example, the tumor susceptibility 101 protein and the ALG-2-interacting protein X) were increased 7 days after ischemia–reperfusion injury in rats, which may point to the underlying mechanisms during the transition of acute kidney injury to renal fibrosis [6]. The authors did not report AQP2 measurements on the first postoperative day in their animal study, whereas in the present study, we focused on early effects on the first postoperative day in kidney transplant recipients. Chan et al. showed that uEV-AQP2 levels might be associated with acute kidney injury in patients with decompensated heart failure [30].

The determination of uEV-AQP2 in the early phase after kidney transplantation may have broad clinical applications. The determination of uEV-AQP in urine obtained 6 to 24 h after transplant may help to identify the extent of allograft damage. Several factors may contribute to allograft damage leading to acute kidney injury. Therefore, it should be noted that higher uEV-AQP2 levels predicted higher rates of acute allograft injury, particularly in recipients who were anuric prior to transplant as well as recipients who received an allograft from a female donor. This may be due to sample sizes. Furthermore, the determination of uEV-AQP2 may help guide the use of diuretics after kidney transplantation. We did not evaluate the abundance of EVs from all tubular cell types, and we cannot exclude that EVs from other cell types, such as intercalated cells, may have a more significant difference between DGF and IGF. SGLT2 and NCC are mainly expressed in the cortical regions of the kidney, whereas AQP2 expression is in the cortical and medullary regions. The kidney medulla regions have low oxygen tension, and we hypothesize that AQP2 EVs are mainly derived from the medullary regions and are released as a response to ischemia.

In conclusion, determining principal cell-specific AQP2 abundance in uEVs early in the first postoperative day quantifies principal cell damage after kidney transplantation and is a non-invasive predictor for acute kidney injury.

## Figures and Tables

**Table 1:** Demographic and clinical characteristics of deceased donor kidney transplant recipients of the discovery cohort who were stratified according to posttransplant acute allograft injury.

	All Patients (N = 16)	Acute Allograft Injury (N = 7)	Immediate Kidney Function (N = 9)	*p*-Value
Age of recipient (years)	48 (43–63)	49 (44–60)	47 (44–67)	0.98
Recipient gender male, N (%)	11 (69%)	5 (71%)	6 (67%)	1.00
Body weight (kg)	78 (68–98)	70 (65–107)	80 (70–98)	0.96
Body mass index (kg/m^2^)	24.7 (23.0–30.9)	24.3 (22.3–31.9)	25.0 (23.3–29.5)	0.80
Systolic blood pressure (mmHg)	146 (120–177)	126 (110–165)	150 (135–177)	0.42
Diastolic blood pressure (mmHg)	80 (76–90)	80 (72–85)	81 (78–90)	0.42
Hypertension, N (%)	15 (94%)	7 (100%)	8 (89%)	1.00
Smoking, N (%)	4 (25%)	1 (14%)	3 (33%)	0.48
Cardiovascular disease, N (%)	2 (13%)	1 (14%)	1 (11%)	1.00
Dialysis, N (%)				1.00
Hemodialysis	12 (75%)	5 (71%)	7 (78%)	
Peritoneal dialysis	3 (19%)	2 (29%)	1 (11%)	
Preemptive	1 (6%)	0 (0%)	1 (11%)	
Dialysis vintage (months)	18 (12–36)	12 (12–28)	24 (10–46)	0.74
Anuric before transplant, N (%)	7 (44%)	4 (57%)	3 (33%)	0.61
Medication, N (%) Angiotensin converting enzyme inhibitor	3 (19%)	2 (29%)	1 (11%)	0.50
Angiotensin receptor blocker	3 (19%)	1 (14%)	2 (22%)	1.00
Beta receptor blocker	6 (38%)	1 (14%)	5 (56%)	0.14
Diuretics	7 (44%)	3 (43%)	4 (44%)	1.00
Age of the donor (years)	53 (48–68)	67 (53–71)	49 (31–56)	0.05
Donor gender female, N (%)	6 (38%)	2 (29%)	4 (44%)	0.63
Number of HLA mismatches (range) ^c^	4 (4–5)	4 (4–5)	5 (4–5)	0.47
Cold ischemic time (min)	810 (658–1155)	900 (600–1155)	810 (723–1073)	0.93
Plasma creatinine pretransplant (µmol/L)	713 (598–960)	940 (749–1025)	924 (545–713)	0.12
Induction therapy, N (%) Basiliximab Thymoglobulin	14 (88%) 2 (12%)	6 (86%) 1 (14%)	8 (89%) 1 (11%)	1.00 1.00

Acute allograft injury was defined by at least one dialysis within the first week after transplantation. HLA denotes human leukocyte antigen. Continuous data are presented as median (interquartile range). Categorical data are presented as numbers (percent). Groups containing continuous data were compared using the Mann-Whitney test, whereas groups containing categorical data were compared using Fisher’s exact test or Chi-square test, as appropriate.

**Table 2:** Demographic and clinical characteristics of deceased donor kidney transplant recipients of the validation cohort who were stratified according to posttransplant acute allograft injury.

	All Patients (N = 82)	Acute Allograft Injury (N = 29)	Immediate Kidney Function (N = 53)	*p*-Value
Age of recipient (years)	50 (43–57)	54 (43–57)	50 (41–58)	0.54
Recipient gender male, N (%)	43 (32%)	17 (59%)	26 (49%)	0.49
Body weight (kg)	71 (61–85)	73 (64–82)	70 (60–85)	0.75
Body mass index (kg/m^2^)	24.8 (21.2–27.5)	25.0 (22.1–27.7)	24.4 (20.8–274)	0.72
Systolic blood pressure (mmHg)	141 (130–160)	146 (140–160)	140 (130– 160)	0.55
Diastolic blood pressure (mmHg)	87 (79–92)	90 (80–97)	82 (75–90)	0.30
Cause of kidney disease, N (%)				0.37
Glomerulo- nephritis	36 (44%)	10 (34%)	26 (49%)	
Diabetes mellitus	13 (16%)	4 (14%)	9 (17%)	
Hypertension	9 (11%)	5 (17%)	4 (8%)	
Interstitial nephritis	4 (5%)	3 (10%)	1 (2%)	
Polycystic kidney disease	9 (11%)	3 (10%)	6 (11%)	
Other / Unknown	11 (13%)	4 (14%)	7 (13%)	
Hypertension, N (%)	63 (77%)	25 (86%)	38 (72%)	0.17
Smoking, N (%)	11 (13%)	3 (10%)	8 (15%)	0.74
Cardiovascular disease, N	18 (22%)	10 (34%)	8 (15%)	0.05
(%)				
Coronary artery	14 (17%)	8 (28%)	6 (11%)	
disease				
Peripheral artery	1 (1%)	1 (3%)	0 (0%)	
disease				
Stroke	3 (4%)	1 (3%)	2 (4%)	
Dialysis, N (%)				0.74
Hemodialysis	72 (88%)	25 (86%)	47 (89%)	
Peritoneal dialysis	10 (12%)	4 (14%)	6 (11%)	
Dialysis vintage (months)	25 (11–43)	35 (12–61)	23 (11–33)	0.11
Anuric before transplant, N (%)	29 (35%)	14 (48%)	15 (28%)	0.09
Medication, N (%) Angiotensin converting enzyme inhibitor	14 (17%)	5 (17%)	9 (17%)	1.00
Angiotensin receptor blocker	26 (23%)	11 (38%)	15 (28%)	0.45
Beta receptor blocker	40 (49%)	17 (59%)	23 (43%)	0.25
Diuretics	16 (20%)	7 (24%)	9 (17%)	0.56
Age of the donor (years)	47 (37–61)	50 (40–65)	46 (32–57)	0.20
Donor gender female, N (%)	34 (41%)	14 (48%)	20 (38%)	0.48
Number of HLA mismatches (range) ^c^	4 (3–4)	4 (3–4)	3 (3–4)	0.31
Cold ischemic time (min)	840 (734–1020)	960 (780–1140)	806 (720–960)	0.02
Plasma creatinine pretransplant, (µmol/L)	756 (636–933)	884 (729–1053)	730 (614–885)	0.01
Induction therapy, N (%) Basiliximab Thymoglobulin	77 (94%) 3 (4%)	25 (86%) 1 (2%)	52 (98%) 2 (3%)	0.05 0.28

Acute allograft injury was defined by at least one dialysis within the first week after transplantation. HLA denotes human leukocyte antigen.Continuous data are presented as median (interquartile range). Categorical data are presented as numbers (percent). Groups containing continuous data were compared using the Mann-Whitney test, whereas groups containing categorical data were compared using Fisher’s exact test or Chi-square test, as appropriate.

## Data Availability

All data and analyses are given in the present manuscript.

## Supplementary Materials

The following supporting information can be downloaded at: https://www.mdpi.com/article/10.3390/biom14091124/s1, Supplementary File S1: Original Western Blots Images.
