# Supplementary figures and images for: Urinary Extracellular Vesicles for Non-Invasive Quantification of Principal Cell Damage in Kidney Transplant Recipients
Source: Biomolecules. 2024 Sep 5;14(9):1124. doi: 10.3390/biom14091124 (PMC11430813; doi:10.3390/biom14091124)

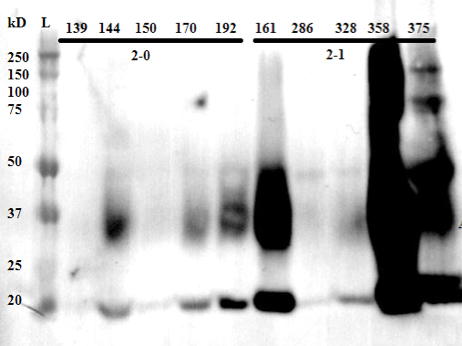

Supplement: Supplementary file 1 [file biomolecules-14-01124-s001.zip › aqp2_d1.jpg]

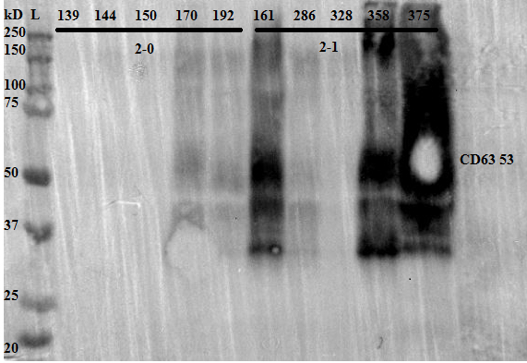

Supplement: Supplementary file 1 [file biomolecules-14-01124-s001.zip › CD63.jpg]

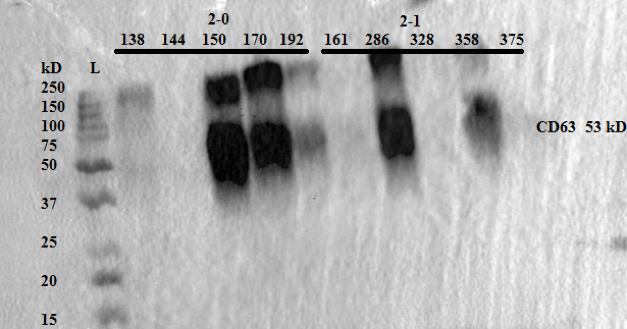

Supplement: Supplementary file 1 [file biomolecules-14-01124-s001.zip › CD63_d29.jpg]

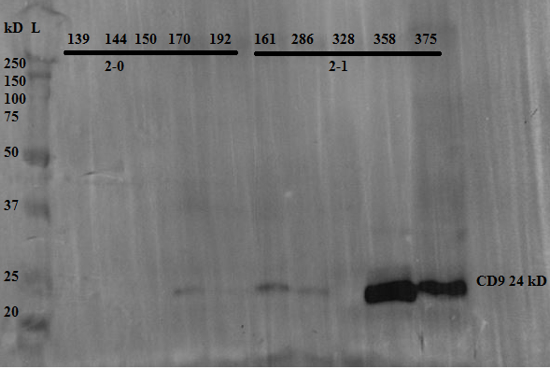

Supplement: Supplementary file 1 [file biomolecules-14-01124-s001.zip › cd9.jpg]

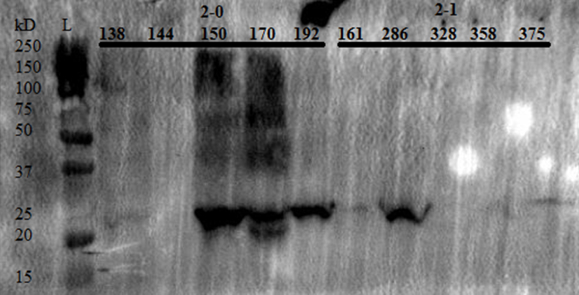

Supplement: Supplementary file 1 [file biomolecules-14-01124-s001.zip › cd9_d29.jpg]

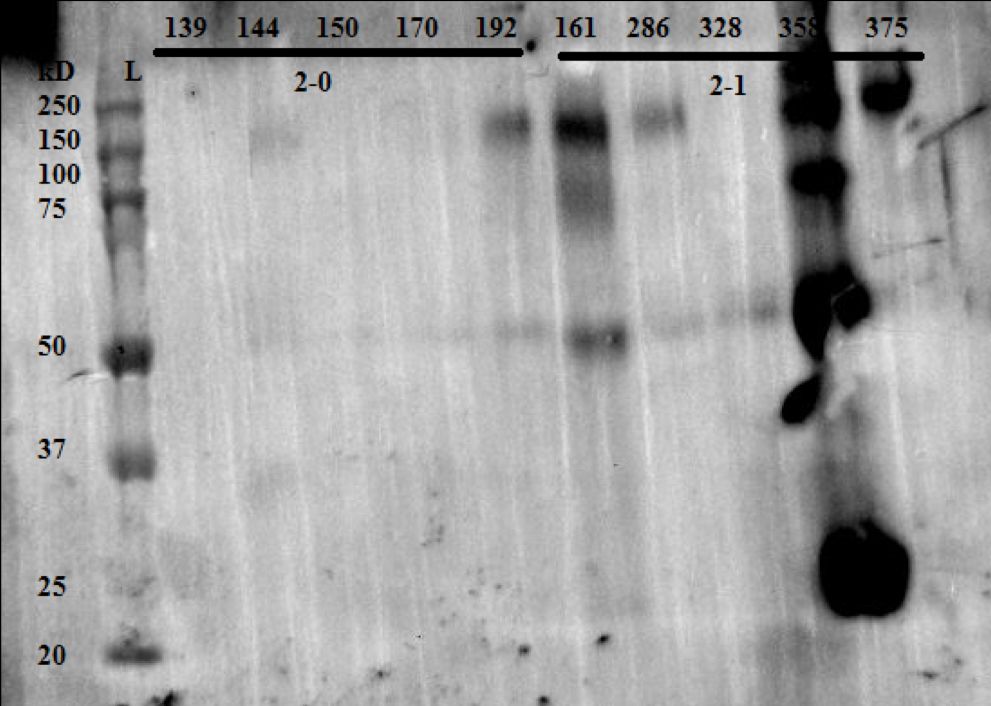

Supplement: Supplementary file 1 [file biomolecules-14-01124-s001.zip › NCC_d1.jpg]

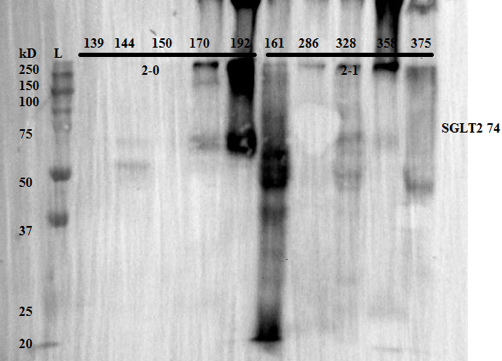

Supplement: Supplementary file 1 [file biomolecules-14-01124-s001.zip › sglt2_d1.jpg]
